# In-depth method assessments of differentially expressed protein detection for shotgun proteomics data with missing values

**DOI:** 10.1038/s41598-017-03650-8

**Published:** 2017-06-13

**Authors:** Jinxia Wang, Liwei Li, Tao Chen, Jie Ma, Yunping Zhu, Jujuan Zhuang, Cheng Chang

**Affiliations:** 1 State Key Laboratory of Proteomics, Beijing Proteome Research Center, National Center for Protein Sciences (Beijing), National Engineering Research Center for Protein Drugs, Beijing Institute of Radiation Medicine, Beijing, 102206 P.R. China; 2 Department of Mathematics, Dalian Maritime University, Dalian, 116026 P.R. China; 3 Drug Research and Development Center, Shandong Drug and Food Vocational College, Weihai, 264210 P.R. China

## Abstract

Considering as one of the major goals in quantitative proteomics, detection of the differentially expressed proteins (DEPs) plays an important role in biomarker selection and clinical diagnostics. There have been plenty of algorithms and tools focusing on DEP detection in proteomics research. However, due to the different application scopes of these methods, and various kinds of experiment designs, it is not very apparent about the best choice for large-scale proteomics data analyses. Moreover, given the fact that proteomics data usually contain high percentage of missing values (MVs), but few replicates, a systematic evaluation of the DEP detection methods combined with the MV imputation methods is essential and urgent. Here, we analyzed a total of four representative imputation methods and five DEP methods on different experimental and simulated datasets. The results showed that (i) MV imputation could not always improve the performances of DEP detection methods and the imputation effects differed in the missing value percentages; (ii) the DEP detection methods had different statistical powers affected by the percentage of MVs. Two statistical methods (*i.e*. the empirical Bayesian random censoring threshold model, and the significance analysis of microarray) performed better than the other evaluated methods in terms of accuracy and sensitivity.

## Introduction

Due to the rapid improvement of high resolution mass spectrometers, the focus of proteomics research is changing from qualitative to quantitative analyses^1^. It is of great significance to accurately determine the protein expression levels and detect DEPs in different experimental conditions (groups or samples) in quantitative proteomics. As one of the major prerequisites of biomarker selection, DEP detection plays an important role in advancing medical researches such as the early diagnosis of disease and prognosis of treatment interventions^2, 3^. Many statistical methods and tools have been proposed to analyze proteins from different biological states. According to the general factions of statistical theory, DEP detection methods for proteomics data can be roughly divided into two categories: the methods based on the Classic Statistics School or the Bayesian School, and the other methods directly borrowed from other fields such as genomics and transcriptomics^4^.

However, there are few evaluations about the detection methods on different kinds of quantitative proteomics data, especially for those with MVs. Pursiheimo *et al*.^5^ evaluated several commonly used statistical methods for DEP detection, but neglecting the influence of MVs in the proteomics data. The primary reasons why a peptide is typically not observed in mass spectrometry (MS) analyses include low abundance, poor ionization, and random sampling in shotgun proteomics. According to the different missing mechanism, MVs can be broadly classified into three categories^6^: missing completely at random (MCAR), missing at random (MAR), and missing not at random (MNAR). Generally, imputation methods can be divided into single imputation and multiple imputation according to the number of estimated values used. Single imputation can be further classified into deterministic imputation and random imputation. Multiple imputation contains two major algorithms: the multivariate normal model^7^ and the univariate chained equations model^8^.

It is often known that MVs can lead to incomplete quantitative proteomic results, which has a detrimental effect on DEP detection. Even if some DEP detection methods might be applied to a dataset containing MVs, their statistical powers tend to be limited by the wide dynamic percentage of MVs in the proteomics data. Webb-Robertson *et al*.^9^ has reviewed some selected imputation methods for label-free quantitative proteomics, but the influences of these imputation strategies on the subsequent DEP detection algorithms were not considered.

Here, a systematic evaluation of DEP detection methods and MV imputation methods was performed for different experimental designs containing different replicates and MV percentages. Our aim is to evaluate the statistical powers of DEP detection methods before and after MV imputation. Specifically, four popular imputation methods and five representative DEP detection methods were comprehensively evaluated on two experimental datasets and nine simulated datasets to answer three scientific questions: (1) What’s the maximum MV percentage of a dataset that imputation methods can handle? (2) To what extent, the imputation could affect the performances of the DEP detection methods? (3) Among the combinations of MV imputation and DEP detection methods, which one is more suitable for proteomics data?

## Results

### Evaluation of the DEP detection methods

**Figure 1:**
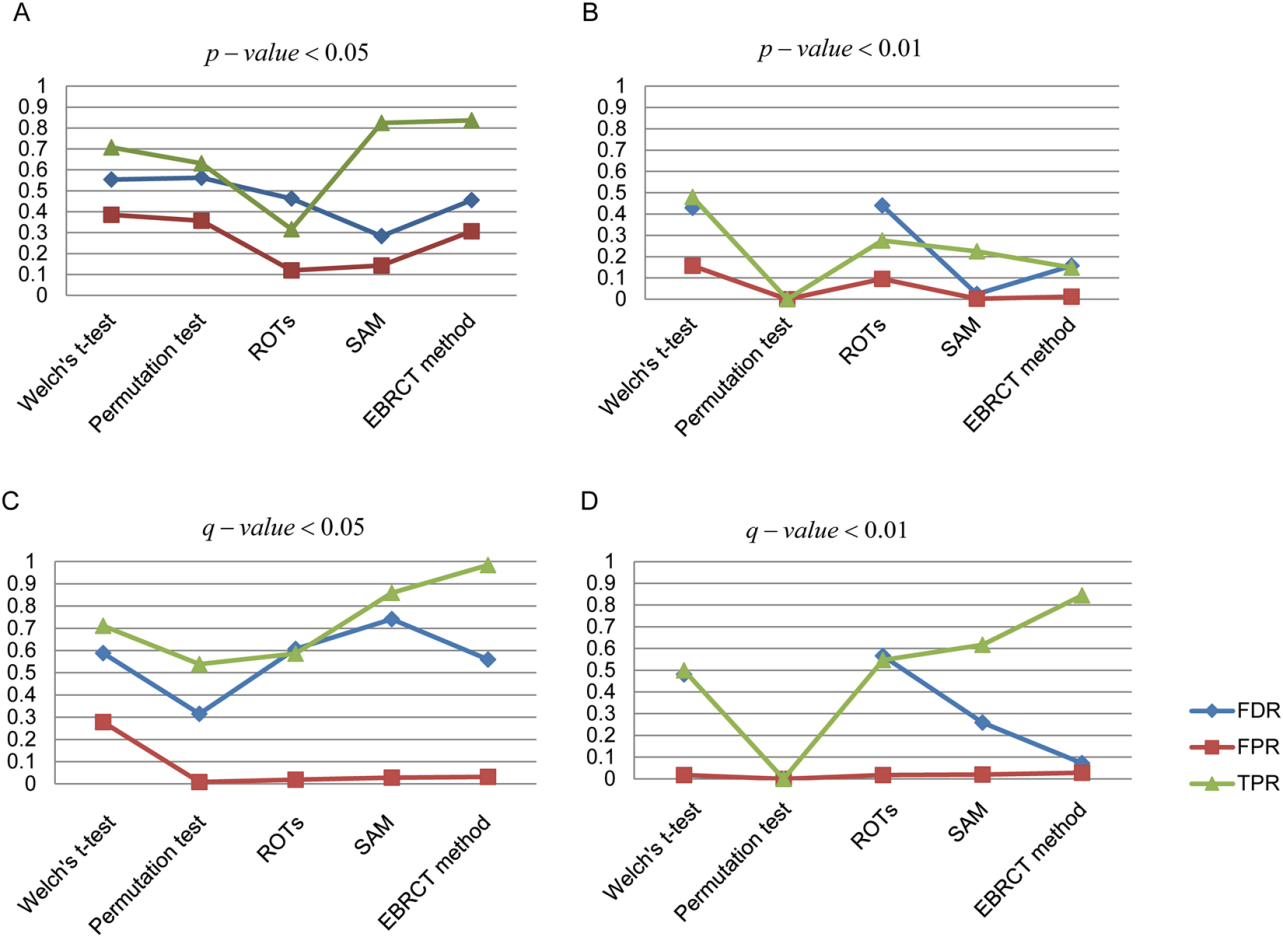
Line charts of FDR, FPR and TPR changes for five different DEP detection methods. (**A**) p-value < 0.05 when using D1, (**B**) p-value < 0.01 when using D1, (**C**) q-value < 0.05 when using D2, (**D**) q-value < 0.01 when using D2.

We calculated p-values for both HeLa and *E*. *coli* proteins using the above mentioned five statistical methods on dataset D1 and based on the definitions of the statistical indexes in Supplementary Table S1, the detailed values of TPR, FPR and FDR were determined using two p-value cutoffs (0.05 and 0.01). An ideal DEP detection method should have a high TPR (sensitivity), but a low FPR and FDR, which is hard to get the best of both worlds in practice. As shown in Fig. 1A and B, SAM and EBRCT performed better than the other methods on dataset D1, for they have high TPR values, but relatively small FPR and FDR values, regardless of the p-value cutoff. Note that when the p-value cutoff is 0.01, the numbers of TP and FP in permutation test are both zeros on dataset D1, resulting in the zero value for TPR and FPR.

Besides the two groups situation in dataset D1 which is a common design for DEP detection in quantitative proteomics, there are also many experiments including three or more than three groups for DEP detection. In these cases, multiple hypothesis testing should be applied to control the quality of the results. In dataset D2, we analyzed four adjacent group pairs (A-B, B-C, C-D, and D-E) with the same statistical methods and obtained a p-value (*p*_*ij*_) for the *j* ^*th*^ protein analyzed in the *i* ^*th*^ adjacent group pair. And for the multiple hypothesis testing, we used the Benjamini-Hochberg method^10^ to determine the q-value of every protein. As shown in Fig. 1C and D, the average values of TPR, FPR and FDR of four group pairs using the five statistical methods were calculated with the q-value cutoff 0.05 and 0.01. Similar with the conclusion in Fig. 1A and B, EBRCT and SAM outperformed than the other methods with a high TPR and a low FDR.

**Figure 2:**
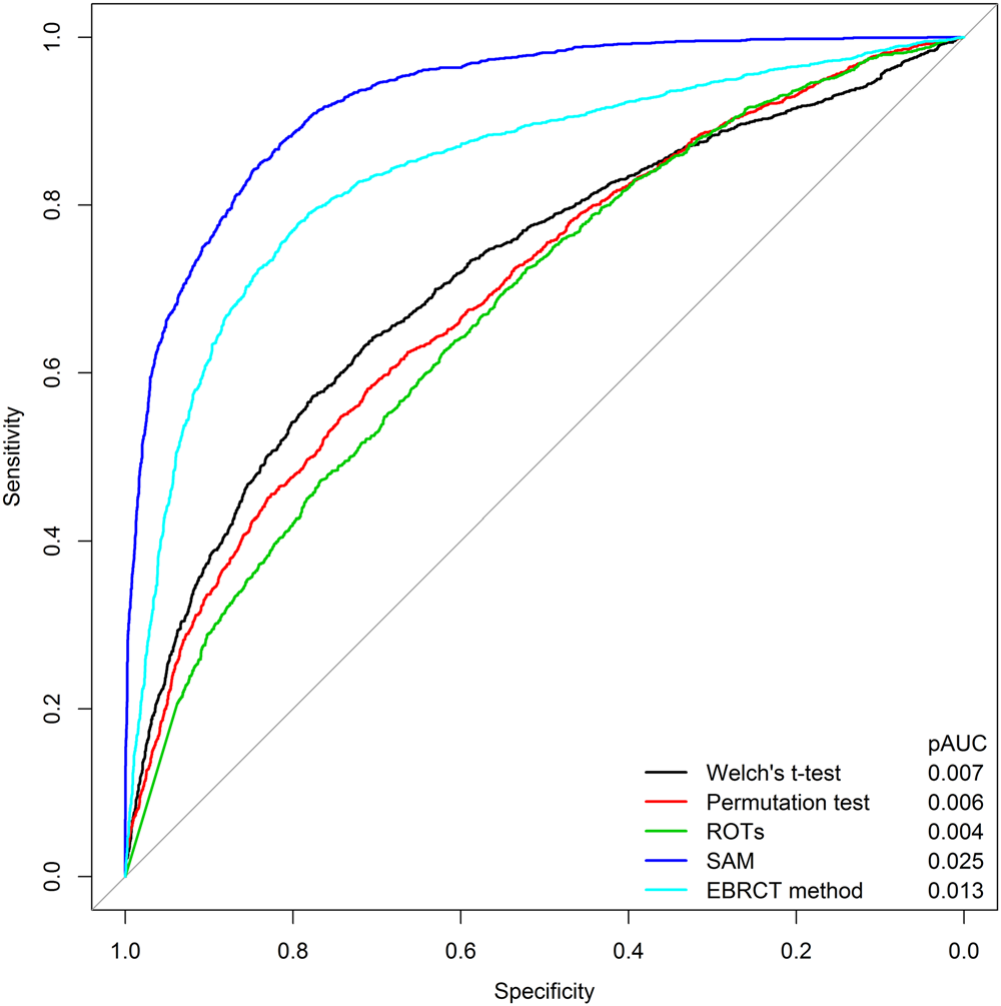
ROC curves of five different DEP detection methods using D1. The pAUC values were listed on the right bottom of this figure.

In practical DEP analyses, we usually focus on the sensitivity of a DEP detection method when the FPR is low, such as the 0.05 cutoff. So to evaluate the practical statistical powers of the five DEP detection methods, we drew the receiver operating characteristic (ROC) curve for every method and calculated the corresponding partial area under curve (pAUC), in which the pAUC was the AUC when the specificity was larger than 0.95 (i.e. FPR < 0.05). As shown in Fig. 2, we found that SAM had the best statistical power, because it had the largest pAUC than the other methods. EBRCT displayed the suboptimal performance, which is in accord with the conclusion in Fig. 1.

Furthermore, we added another two evaluation indexes for the five DEP detection methods: detection rate (DR) and mean rank (MR)^11^ of the UPS1 proteins in D2. DR and MR are measurements of the accuracy and sensitivity of a DEP detection method, respectively. A large DR value indicates a high accuracy, while a small MR value represents a high sensitivity. The detailed computational processes of the two indexes are described as follows. Firstly, we put UPS1 proteins in front of the yeast proteins in the proteins list of D2, and then labeled all proteins using a sequential labeling method. Essentially, the labeled number was the data rank. Secondly, we sorted all proteins in descending order according to p-values and chose the top 32 UPS1 proteins. Finally, we calculated the number (N) and the rank sum (RS) of the true positive proteins. Thus, we calculated the DR and MR as *DR* = *N*/32 and *MR* = *RS*/*N*. As shown in Table 1, EBRCT outperformed the others and SAM showed the suboptimal performance. In summary, the performance of the methods based on the Classic Statistics School (e.g., Welch’s t-test, permutation test, and ROTs) were not so good. SAM and EBRCT performed better than the other methods in terms of specificity and sensitivity on both two experiment datasets D1 and D2.

**Table 1:** Values of detection rate (DR) and mean rank (MR) of five different DEP detection methods in D2 when q-value is smaller than 0.05.

Method	DR	MR
Welch’s t-test	0.485	16.937
Permutation test	0.579	16.989
ROTs	0.523	16.932
SAM	0.664	16.846
EBRCT	0.899	16.264

### Effect of imputation on DEP detection

The high level of MVs in quantitative proteomics data made DEP detection difficult, but fortunately, we can make up it by imputation before performing statistical analyses. To find out whether the imputation can increase the performance of statistical test and find a suitable imputation method, we evaluated the statistical effect for every DEP detection method with/without the four different imputation methods mentioned above.

Before this, to explore the relationship between the imputation time (*m*) and the imputation effect, and determine the best *m* for the multiple imputation method (MuI), we firstly assessed its imputation effect with 57 different imputation times in each of the nine simulated datasets. The 57 imputation times were divided into two parts in our study: a total of 48 continuous integers (continuous integers from 3 to 50), and nine discrete large integers (60, 80, 100, 150, 200, 300, 400, 600, and 800), covering all the possible situations in practical analysis.

**Figure 3:**
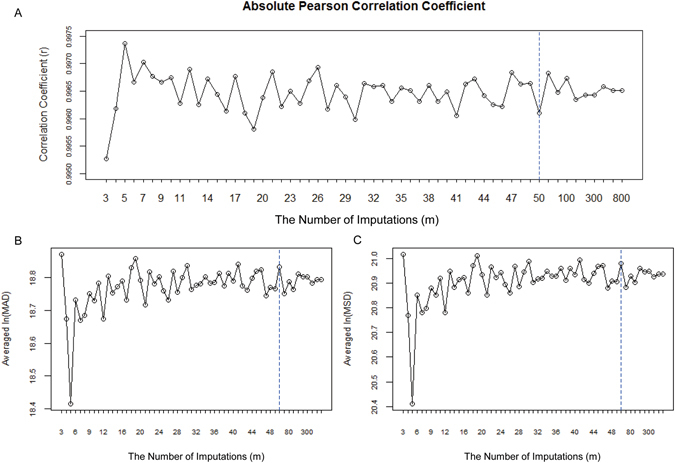
Line charts of absolute Pearson correlation coefficient (**A**) as well as the averaged ln(MAD) (**B**) and ln(MSD) (**C**) in simulated dataset D0(a) with 5% missing value percentage. In each panel, the x axis represents the multiple imputation number (m). In the left side of the dotted line, the m is from 3 to 50 continuously. In the right side, m is 60, 80, 100, 150, 200, 250, 300, 400, 600 and 800, respectively.

The absolute Pearson correlation coefficient (*r*) between the data with/without MuI was calculated in each simulated dataset. Since there were three replicates in all datasets, we computed the correlation coefficient (*r*_1_, *r*_2_, *r*_3_) for each replicate after the first imputation. Next, we took the minimum *r* as the overall correlation coefficient, *r* = min{*r*_1_, *r*_2_, *r*_3_}. The rationale for using the Pearson correlation coefficient rather than the Spearman or Kendall rank correlation coefficients was that the Pearson correlation coefficient is based on the variance and covariance and is therefore sensitive to outliers, which could better show the difference with/without imputation. The absolute Pearson correlation coefficient line charts for nine simulated datasets with different proportions of MVs is shown in Fig. 3A and Supplementary Figs S1A–S8A.

In addition, we added two statistical indexes in our study: mean absolute deviation (MAD, $MAD=({\sum }_{i}^{n}{\rm{\Delta }}{y}_{i})/{n}_{0})$, and modified standard deviation (MSD, $MSD=\sqrt{\frac{1}{{n}_{0}}{{\sum }_{i}^{n}({\rm{\Delta }}{y}_{i})}^{2}}$). For a given replicate, Δ*y*_*i*_ stands for the deviation produced by observations of the *i* ^*th*^ protein before and after imputation, and *n*/*n*_0_/ stands for the number of proteins/proteins with non-zero deviations. For the convenience of drawing the MAD and MSD line charts in each replicate, we calculated the averaged ln(MAD) and ln(MSD) of three replicates to illustrate the averaged imputation effect on overall data (Fig. 3B,C and Supplementary Fig. S1B and C–S8B and C). When the proportion of MVs was 5%, we can find that MuI with five imputation times could reach the highest correlation coefficient and the lowest MAD and MSD. As shown in Fig. 3, the multiple imputation effect differs with the number of imputations and the MV percentage. A larger number of imputations does not equate to a better imputation effect. The best imputation times are different in datasets with different proportions of MVs. Thus, care must be taken when determining the number of imputations when using multiple imputation method in actual research.

**Figure 4:**
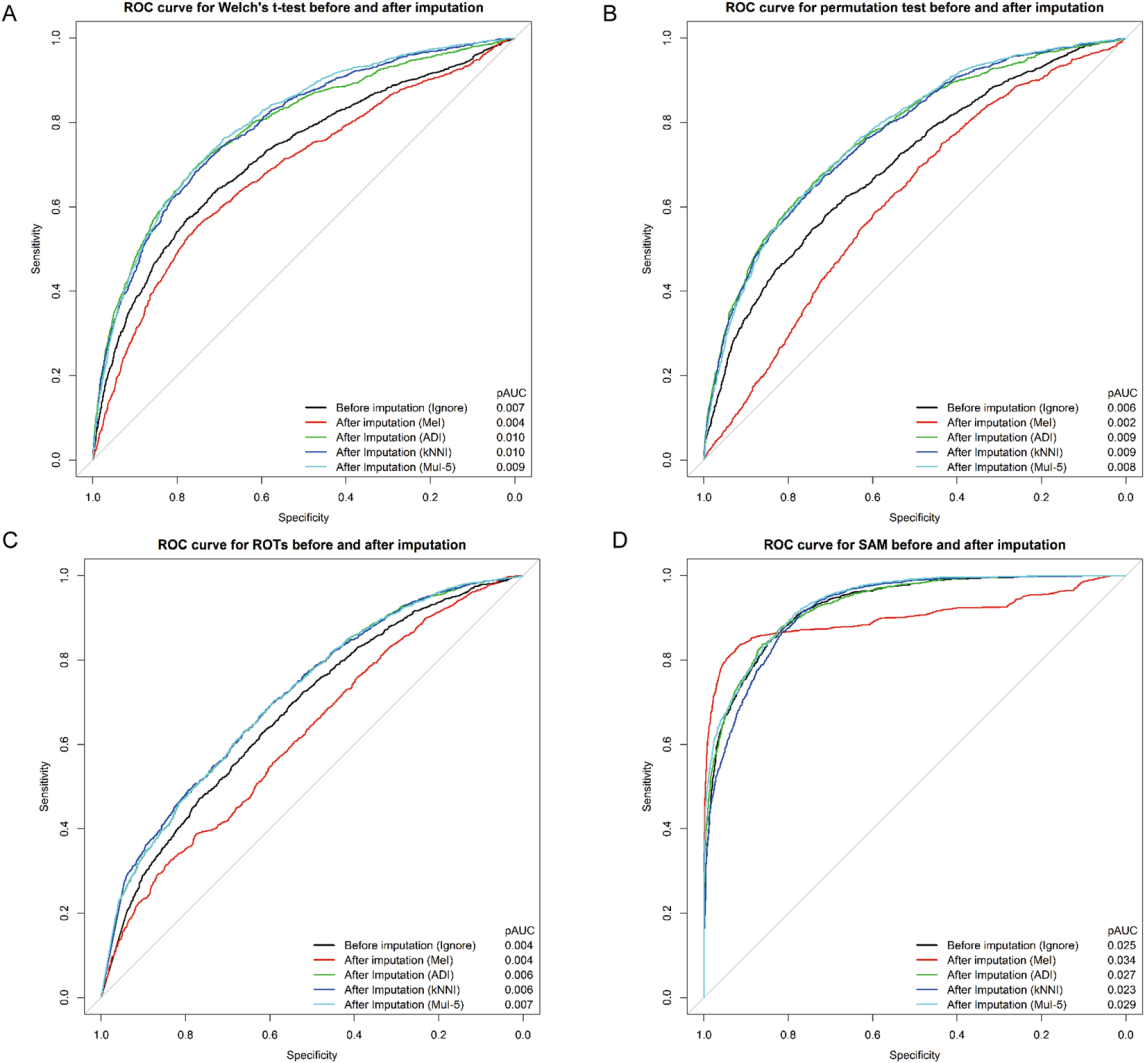
ROC curves for Welch’s t-test (**A**), permutation test (**B**), ROTs (**C**) and SAM (**D**) before and after four different imputation methods in D1. “Ignore” corresponds to the test results not considering the MVs. pAUC values were listed on the right bottom of each panel.

**Figure 5:**
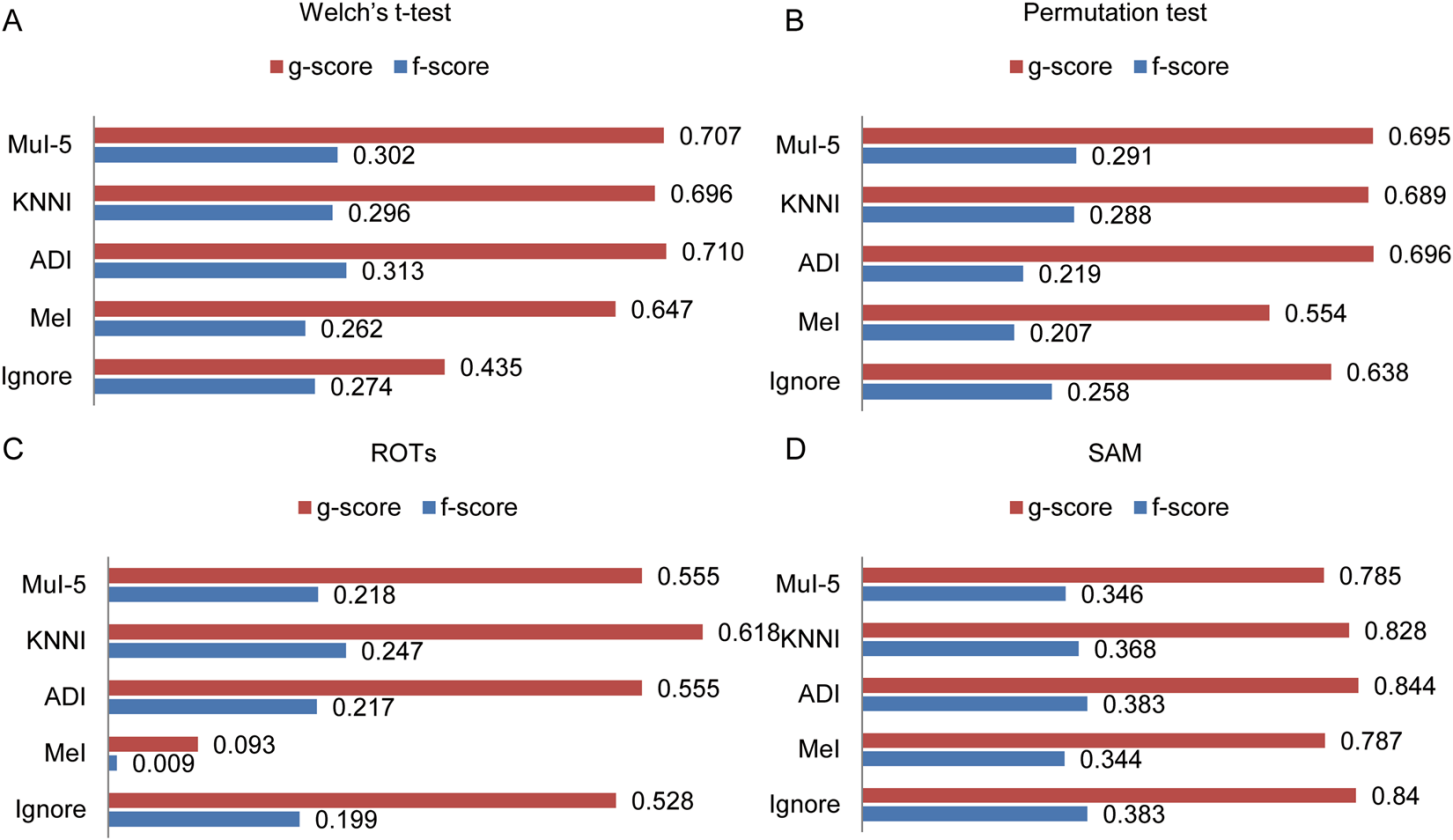
Bar charts of g-score and f-score for Welch’s t-test (**A**), permutation test (**B**), ROTs (**C**) and SAM (**D**) before and after four different imputation methods in D1.

Next, we used MeI, ADI, kNNI and MuI with five imputation times to fill MVs in each group of dataset D1. Then, the four statistical analyses, *i.e*. Welch’s t-test, permutation test, ROTs and SAM were performed to detect DEPs in dataset D1. Note that EBRCT is claimed to be able to handle the data with MVs, thus it is not evaluated here. As shown in Fig. 4, to evaluate the performances of statistical tests before and after imputation, we drew ROC curves, calculated the pAUC and compared them with the corresponding results of Fig. 2. The symbol “Ignore” represents no imputation before DEP detection. Furthermore, the g-score and f-score also were calculated when the p-value cutoff was 0.05 (Fig. 5). The detailed definitions of g-score and f-score are described in Supplementary Table S1. As shown in Figs 4 and 5, most of the imputation methods, except MeI, could improve the performances of all the statistical methods to some extent. Each statistical method has its own suitable imputation method, which can be regarded as an optional means to improve the statistical effect of DEP detection method.

## Conclusions

In quantitative proteomics, the detection of DEPs has become increasingly important. The main objective of our study is to make in-depth assessments for several representative DEP detection methods using two experimental datasets with known differentially expressed proteins and nine simulated datasets with different MV percentages. Firstly, our evaluation demonstrated that SAM and EBRCT showed the relatively better statistical powers than the methods based on the Classical Statistical School (Welch’s t-test, permutation test and ROTs). EBRCT has an advantage that it imputes the missing values at first. SAM is able to perform data de-noise according to the data distribution on the basis of t-test. Hence, both of the two methods own outperformed the other methods. The methods combining the Classic Statistical School and the Bayesian School might be a promising choice.

Secondly, we also explored and discussed the effect of imputation on DEP detection methods. The results indicate that imputation can enhance the performances of some certain statistical methods to some extent. In addition, for multivariate imputation by chained equations, the imputation effect differs with the number of imputations and the proportion of MVs in the dataset. The performance of multivariate imputation by chained equations with five imputation times was the best choice for datasets containing 5% missing values. A large imputation time does not guarantee a good imputation effect.

To conclude, the different experiment designs and data characteristics require different DEP statistical detection methods and imputation methods. Our results are expected to provide helpful insights for the corresponding researchers.

## Methods

### Datasets

There were two experimental datasets (D1 and D2) and nine simulated datasets *i.e*. D0(a)~D0(i) used for systematic evaluation in this study.

The first experimental dataset (D1) was originated from the results of Cox *et al*. paper^12^ which contained two mixed groups: 60 µg HeLa samples combined with two different concentrations (10, 30 µg) of *E. coli* with three technical replicates in either group. All the HeLa proteins were theoretically presented at a ratio of 1:1, while all the *E*. *coli* proteins were at a ratio of 1:3 between the two different groups. The decoy and contaminant proteins are removed before evaluation. Protein abundances were taken from the median normalized LFQ values. Finally, a total of 5325 proteins including 1623 *E. coli* proteins were quantified at least once in either group and kept for future evaluation (Supplementary Table S2).

The second experimental dataset (D2) was from Study 6 *OrbitrapP@65* of the CPTAC (Clinical Proteomic Technology Assessment for Cancer) Network^13^, which consisted of five groups, labeled *A* to *E*, where five different concentrations (0.25, 0.74, 2.2, 6.7 and 20 fmol/µL) of UPS1 (Proteomics Dynamic Range Standard, Sigma-Aldrich) were added to a constant yeast background (60 ng/µL). There were three replicates for every concentration. All the raw data were downloaded from https://cptac-data-portal.georgetown.edu/cptac/public?scope=Phase+I. MaxQuant^14^ (version 1.5.0.25) was used for protein identification and label-free quantification. Using default settings, we searched against the UniProt yeast protein database (version 2013–11) combined with the 48 UPS1 protein sequences. A peptide and protein FDR of 0.01 was applied for quality control in identification and quantification. Finally, 1009 proteins were kept in the evaluation, including 32 UPS1 proteins (Supplementary Table S3). All the protein intensities in datasets D1 and D2 were log-transformed for the next analysis.

Besides the two experimental datasets, we simulated nine datasets with different proportions of MVs (range 5% to 45% with a 5% increment per dataset) based on D1, namely D0(a)~D0(i). Firstly, we randomly selected 1000 proteins from D1 to obtain a complete dataset (D0). All the selected proteins were required to be observed in every experimental condition and replicate. Secondly, we sorted the selected 1000 proteins in each replicate in ascending order in terms of their expression levels. These 3000 observations (1000 proteins × 3 replicates) were divided into grades I, II, and III, which represented the low, medium, high abundant proteins, respectively. We assumed that the protein numbers in the three grades were 3:4:3 to simulate the real situation in proteomics data. Thus, Grades I and III both contained 900 observations (300 proteins × 3 replicates), and grade II included 1200 observations (400 proteins × 3 replicates). Thirdly, we randomly substituted NA (not available, stands for a MV) for all observations in the three grades with an assuming 6:3:1 ratio. For example, if we set the proportion of MVs in the simulated dataset at 5%, the number of NA in all replicates was 150 (1000 proteins × 3 replicates × 5%). The proportion of MVs in grades I, II, and III was 3%, 1.5%, and 0.5% respectively.

### DEP detection methods

In our study, we evaluated five representative statistical methods, *i.e*. Welch’s t-test, permutation test, reproducibility-optimized test statistic (ROTs)^15^, empirical Bayesian random censoring threshold (EBRCT)^16^, and significance analysis of microarray (SAM)^17, 18^.

Welch’s t-test is one of the independent two-sample t-test and often used in proteomics to assess differences in the average expression levels of a given protein between two groups. With an assumption of equal sample size in two groups, the statistic can be simply calculated as $t(i)=\frac{{\bar{x}}_{1}(i)-{\bar{x}}_{2}(i)}{s(i)}$, where ${\bar{x}}_{j}(i)$ is the average expression level of protein *i* in group *j* and *s*(*i*) is the pooled standard error for the expression of the protein $i$ estimated as $s(i)=\sqrt{\frac{1}{n}({s}_{1}^{2}(i)+{s}_{2}^{2}(i))}$. Here ${s}_{j}^{2}(i)$ is the variance of the expression level of protein *i* in group *j* and *n* denotes the replicates in group *j*. Unlike the student’s t-test, the degree of freedom of Welch’s t-test should be calculated as $df(i)=\frac{n-1}{n}({s}_{1}^{2}(i)+{s}_{2}^{2}(i))$. It should be noted that data should be normally or logarithmically distributed when using t-test. Hence, Welch’s t-test was performed on log-transformed data (natural logarithm, the same as below).

Permutation test is a non-parametric statistical test, which can be appropriate for data with small sample size and unknown sample distribution. It is a compute-intensive analysis that draws statistical inferences according to full (or random) permutation of the sample. In our study, we performed this test using the *oneway_test* function in the R package “coin”.

ROTS is a reproducibility-optimization procedure that selects a protein ranking statistic for a given data by maximizing the reproducibility of the detections among a family of modified t-statistic as ${d}_{\alpha }(i)=\frac{{\bar{x}}_{1}(i)-{\bar{x}}_{2}(i)}{{\alpha }_{1}s(i)+{\alpha }_{0}}$, *α*_0_ ≥ 0, *α*_1_∈{0, 1}. The parameters *α*_0_ and *α*_1_ specify the statistic, and the optimal combination of these parameters is determined by reproducibility maximization. Importantly, the ROTs statistic does not rely on any parametric assumptions. In our study, we performed this test using the *ROTS* function in the R package “ROTS”, and set the permutation times *B* as 500, the largest length of protein list *k* as 1000.

The above three statistical methods belong to the Classical Statistical School, and we also evaluated the EBRCT method, which is an typical algorithm from the Bayesian School. It creates a statistical model for each protein with missing quantitative information, as well as calculates the mean and standard deviation (SD) of the expression level of the target protein in any two adjacent groups. The detailed description can be found in Koopmans’s paper^16^.

SAM is a popular method originally designed for genomics. It calculates a score to each protein, based on the difference in its average abundance between two groups. The SAM statistic is calculated as $d(i)=\frac{{\bar{x}}_{1}(i)-{\bar{x}}_{2}(i)}{s(i)+{s}_{0}}$, which is similar to the t statistic. To make the coefficient of variation of *d*(*i*) across all proteins approximately constant, a small positive value *s*_0_ (a percentile of the standard error values)is added to the denominator. Based on the premise of a lower FDR, this method calculates the p-value and Δ value for each gene in any two different groups. In our study, we performed SAM using the R package “samr”.

### MV imputation methods

In our study, three representative single imputation methods, *i.e*. mean imputation (MeI), k nearest neighbor imputation (kNNI)^19^, abundance distribution-based imputation (ADI), and one multiple imputation method, *i.e*. multivariate imputation by chained equations (MuI)^20^ were evaluated on dataset D1.

MeI and kNNI belong to the category of deterministic imputation methods. MeI replaces a missing value with the mean of observations coming from the target protein or technical replicate, which offers the benefit of not changing the mean of the target protein or the technical replicate. This imputation method consists of row mean imputation and column mean imputation. kNNI also uses the mean of the observations, but this method considers k proteins that are adjacent to the target protein. Here, we set the number of neighbors (k) to 10 by default. The weakness of this imputation method is the complexity when calculating the Euclidean metric between proteins. In our study, we used the R package “impute” for kNNI.

ADI is a randomized imputation method based on abundance distribution to impute MVs. In this study, we firstly sorted observed proteins based on the abundance in ascending order for each replicate. Since most of the absent peptides were low-abundant, we calculated the mean and SD (*μ*_*j*_ and *σ*_*j*_) of the top 25% abundant proteins, in which *j* represents the *j* ^*th*^ replicate (1~3). Secondly, we took the normal distribution as a benchmark function, *N*(*μ*_*j*_, *σ*_*j*_), and randomly extracted positive numbers from this benchmark function to impute MVs for the *j* ^*th*^ replicate. Finally, to be more robust, we repeated the imputation processes 100 times and considered the average as the final result.

As a typical method for multiple imputation, MuI was implemented using the R package “mice”. The detailed computational procedure includes: (a) estimating a certain MV (*x*) by a regression equation and observation; (b) sampling a value (*δ*) randomly from the distribution of residual errors; and (c) using the sum of these two values (*x* + δ) as the predicted value for the target missing value.

## Electronic supplementary material

Supplementary information

Supplementary Table S2

Supplementary Table S3

## References

[1] Cox J, Mann M (2011). Quantitative, high-resolution proteomics for data-driven systems biology. Annu Rev Biochem.

[2] Mischak H (2010). Recommendations for biomarker identification and qualification in clinical proteomics. Sci Transl Med.

[3] Puntmann VO (2009). How-to guide on biomarkers: biomarker definitions, validation and applications with examples from cardiovascular disease. Postgrad Med J.

[4] Wang JX (2015). Statistical Strategies for Selection of Differentially Expressed Proteins Based on Mass Spectrometry Technology. Scientia Sinica Vitae.

[5] Pursiheimo A (2015). Optimization of Statistical Methods Impact on Quantitative Proteomics Data. J Proteome Res.

[6] Little, R. J. A. & Rubin, D. B. *Statistical Analysis with Missing Data*, 24–40 (John Wiley & Sons, Inc., 2002).

[7] J. L., S. *Analysis of Incomplete Multivariate Data*, *C&H/CRC Monographs on Statistics & Applied Probability* (Chapman and Hall/CRC, 1997).

[8] Little RJ (1988). Missing-data adjustments in large surveys. Journal of Business & Economic Statistics.

[9] Webb-Robertson BJ (2015). Review, evaluation, and discussion of the challenges of missing value imputation for mass spectrometry-based label-free global proteomics. J Proteome Res.

[10] Benjamini Y, Hochberg Y (1995). Controlling the False Discovery Rate: A Practical and Powerful Approach to Multiple Testing. Journal of the Royal Statistical Society.

[11] Shan WJ, Tong CF, Shi JS (2008). [Comparison of statistical methods for detecting differential expression in microarray data]. Yi chuan = Hereditas.

[12] Cox J (2014). Accurate proteome-wide label-free quantification by delayed normalization and maximal peptide ratio extraction, termed MaxLFQ. Mol Cell Proteomics.

[13] Tabb DL (2010). Repeatability and reproducibility in proteomic identifications by liquid chromatography-tandem mass spectrometry. J Proteome Res.

[14] Cox J, Mann M (2008). MaxQuant enables high peptide identification rates, individualized p.p.b.-range mass accuracies and proteome-wide protein quantification. Nat Biotechnol.

[15] Elo LL, Filen S, Lahesmaa R, Aittokallio T (2008). Reproducibility-optimized test statistic for ranking genes in microarray studies. IEEE/ACM transactions on computational biology and bioinformatics.

[16] Koopmans F, Cornelisse LN, Heskes T, Dijkstra TM (2014). Empirical Bayesian random censoring threshold model improves detection of differentially abundant proteins. J Proteome Res.

[17] Tusher VG, Tibshirani R, Chu G (2001). Significance analysis of microarrays applied to the ionizing radiation response. Proceedings of the National Academy of Sciences of the United States of America.

[18] Larsson O, Wahlestedt C, Timmons JA (2005). Considerations when using the significance analysis of microarrays (SAM) algorithm. BMC Bioinformatics.

[19] Batista, G. E. & Monard, M. C. A study of K-nearest neighbour as an imputation method. *Proceedings of the Second International Conference on Hybrid Intelligent Systems* **7**, 251–260 (2002).

[20] White IR, Royston P, Wood AM (2011). Multiple imputation using chained equations: issues and guidance for practice. Statistics in Medicine.

