# Supplementary material for: In-depth method assessments of differentially expressed protein detection for shotgun proteomics data with missing values
Source: Sci Rep. 2017 Jun 13;7:3367. doi: 10.1038/s41598-017-03650-8 (PMC5469784; doi:10.1038/s41598-017-03650-8)
Supplement: Supplementary file 1 — Supplementary information [file 41598_2017_3650_MOESM1_ESM.pdf]

In-depth method assessments of differentially expressed protein detection for shotgun proteomics data with missing values

Jinxia Wang[1,2$*], Liwei Li[1*], Tao Chen[1], Jie Ma[1], Yunping Zhu[1], Jujuan Zhuang[2], Cheng Chang[1]

[1]*State Key Laboratory of Proteomics, Beijing Proteome Research Center, National Center for Protein Sciences (Beijing), National Engineering Research Center for Protein Drugs, Beijing Institute of Radiation Medicine, Beijing, 102206, P. R. China*

[2]*Department of Mathematics, Dalian Maritime University, Dalian, 116026, P.R. China.*

[$]*Present address: Drug Research and Development Center, Shandong Drug and Food Vocational College, Weihai, 264210, P.R. China.*

[*]*These authors contributed equally to this work. Correspondence and requests for materials should be addressed to C.C. (email: 1987ccpacer@163.com) or J.Z. (email: jjzhuang@dlmu.edu.cn)*

Supplementary Figures and Tables

List of Figures

Figure S1. Line charts of absolute Pearson correlation coefficient as well as the averaged ln(MAD) and ln(MSD) in simulated dataset D0(b) with 10% missing value percentage.

Figure S2. Line charts of absolute Pearson correlation coefficient as well as the averaged ln(MAD) and ln(MSD) in simulated dataset D0(c) with 15% missing value percentage.

Figure S3. Line charts of absolute Pearson correlation coefficient as well as the averaged ln(MAD) and ln(MSD) in simulated dataset D0(d) with 20% missing value percentage.

Figure S4. Line charts of absolute Pearson correlation coefficient as well as the averaged ln(MAD) and ln(MSD) in simulated dataset D0(e) with 25% missing value percentage.

Figure S5. Line charts of absolute Pearson correlation coefficient as well as the averaged ln(MAD) and ln(MSD) in simulated dataset D0(f) with 30% missing value percentage.

Figure S6. Line charts of absolute Pearson correlation coefficient as well as the averaged ln(MAD) and ln(MSD) in simulated dataset D0(g) with 35% missing value percentage.

Figure S7. Line charts of absolute Pearson correlation coefficient as well as the averaged ln(MAD) and ln(MSD) in simulated dataset D0(h) with 40% missing value

percentage.

Figure S8. Line charts of absolute Pearson correlation coefficient as well as the averaged ln(MAD) and ln(MSD) in simulated dataset D0(i) with 45% missing value percentage.

List of Tables

Table S1. Definitions of the indices used to evaluate the performances of DEP detection methods.

Table S2. Protein quantification results of experimental dataset D1 using MaxQuant.

Table S3. Protein quantification results of experimental dataset D2 using MaxQuant.

Figure S1. Line charts of absolute Pearson correlation coefficient as well as the averaged ln(MAD) and ln(MSD) in simulated dataset D0(b) with 10% missing value percentage.

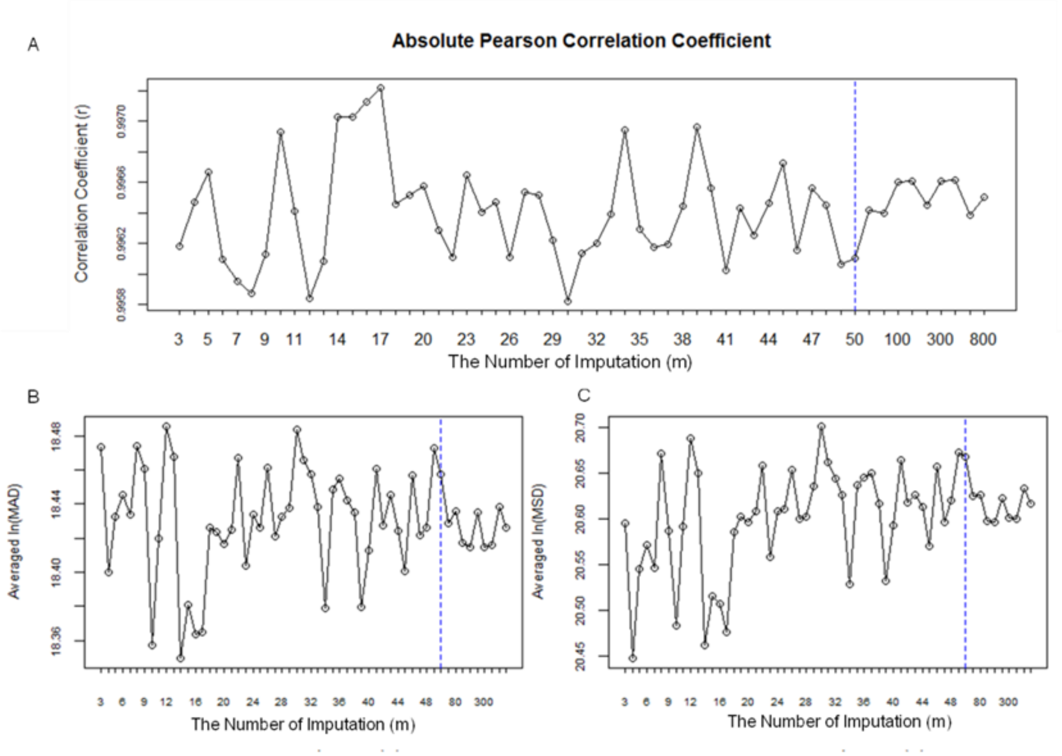

Figure S2. Line charts of absolute Pearson correlation coefficient as well as the averaged ln(MAD) and ln(MSD) in simulated dataset D0(c) with 15% missing value percentage.

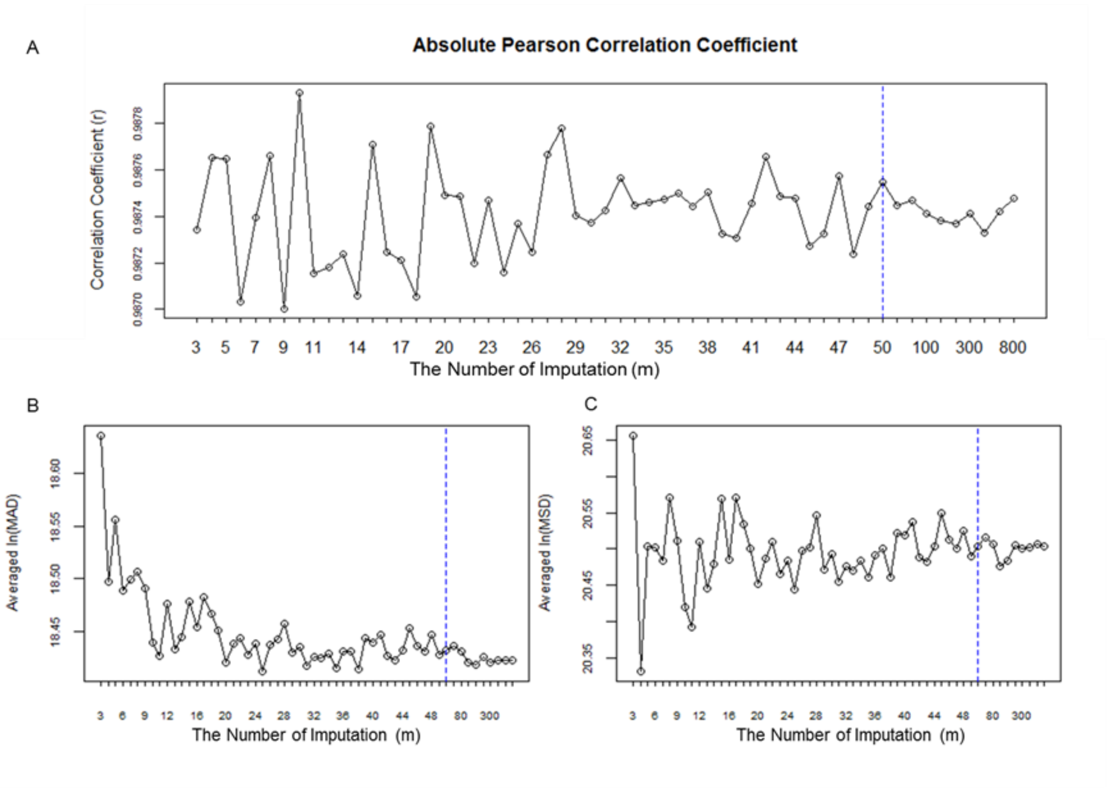

Figure S3. Line charts of absolute Pearson correlation coefficient as well as the averaged ln(MAD) and ln(MSD) in simulated dataset D0(d) with 20% missing value percentage.

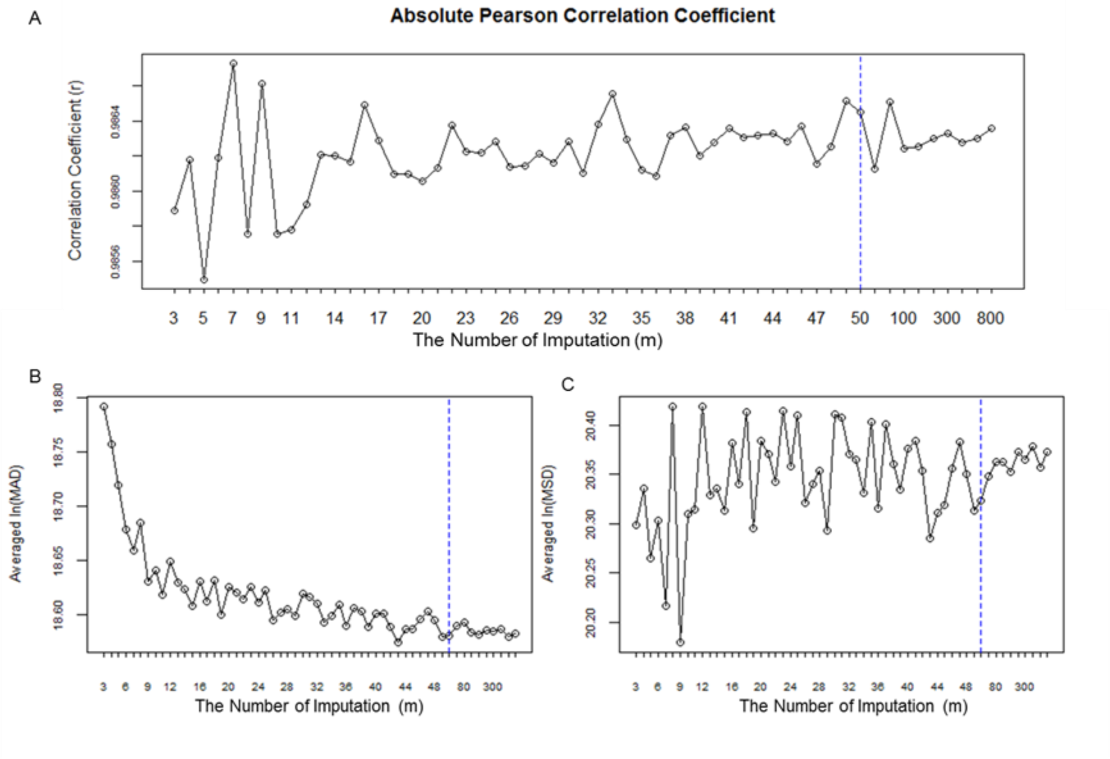

Figure S4. Line charts of absolute Pearson correlation coefficient as well as the averaged ln(MAD) and ln(MSD) in simulated dataset D0(e) with 25% missing value percentage.

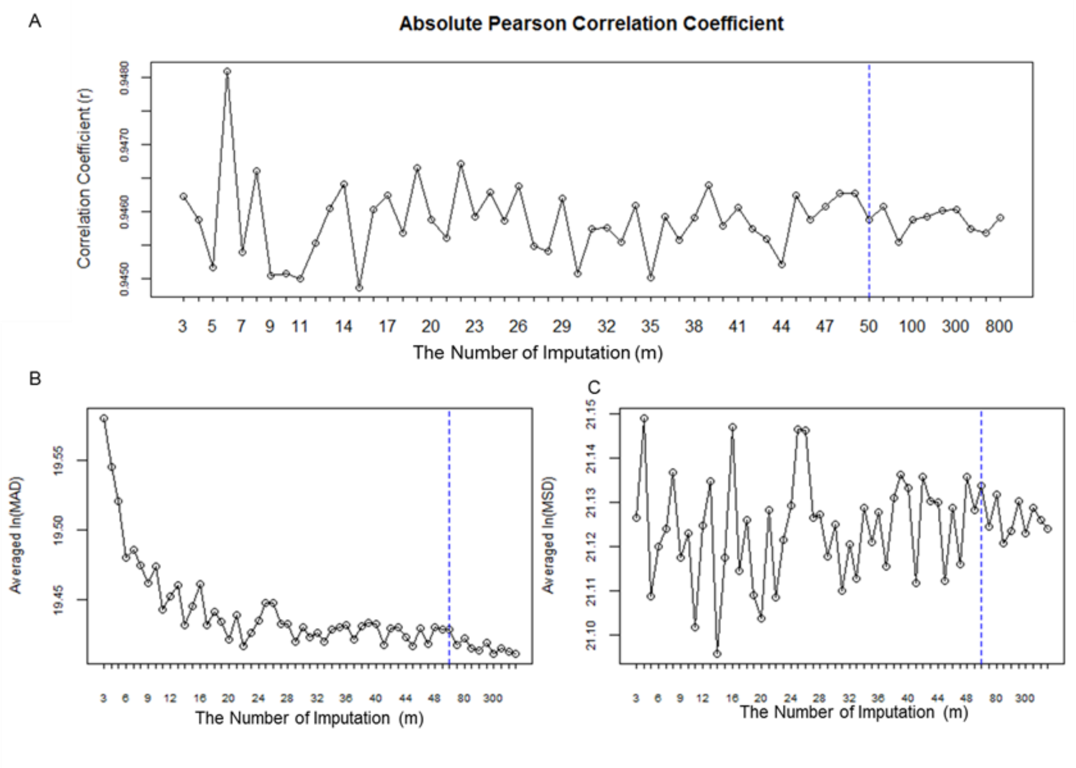

Figure S5. Line charts of absolute Pearson correlation coefficient as well as the averaged ln(MAD) and ln(MSD) in simulated dataset D0(f) with 30% missing value percentage.

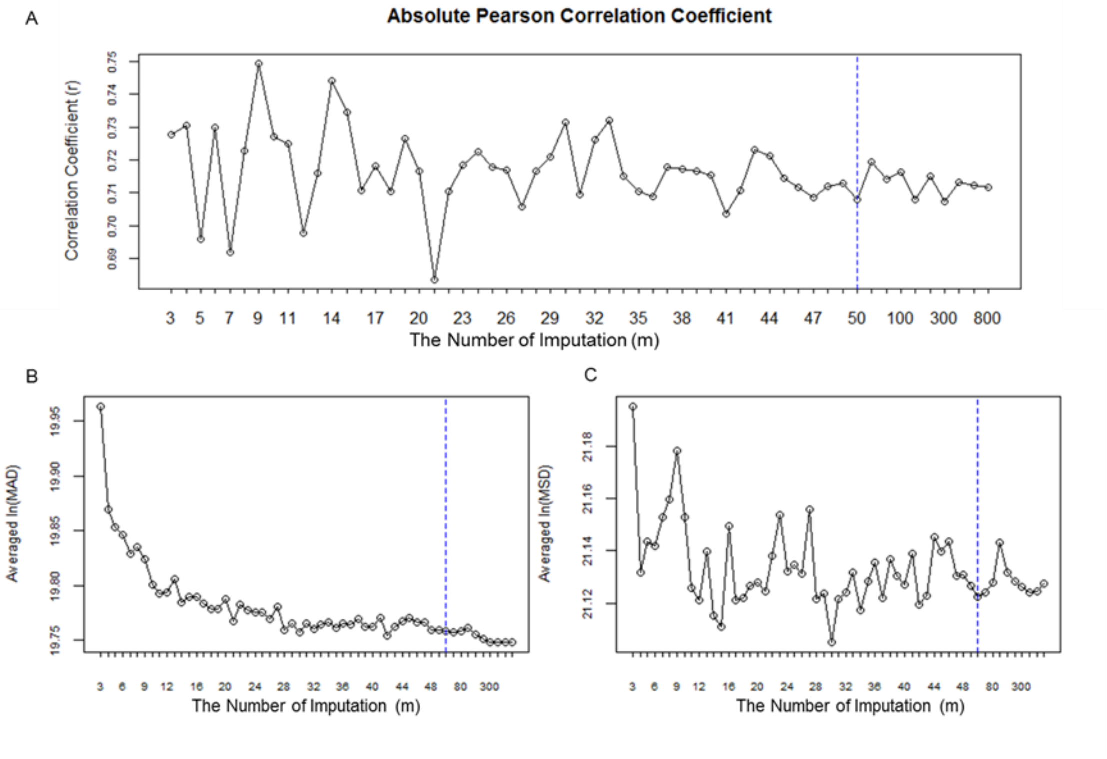

Figure S6. Line charts of absolute Pearson correlation coefficient as well as the averaged ln(MAD) and ln(MSD) in simulated dataset D0(g) with 35% missing value percentage.

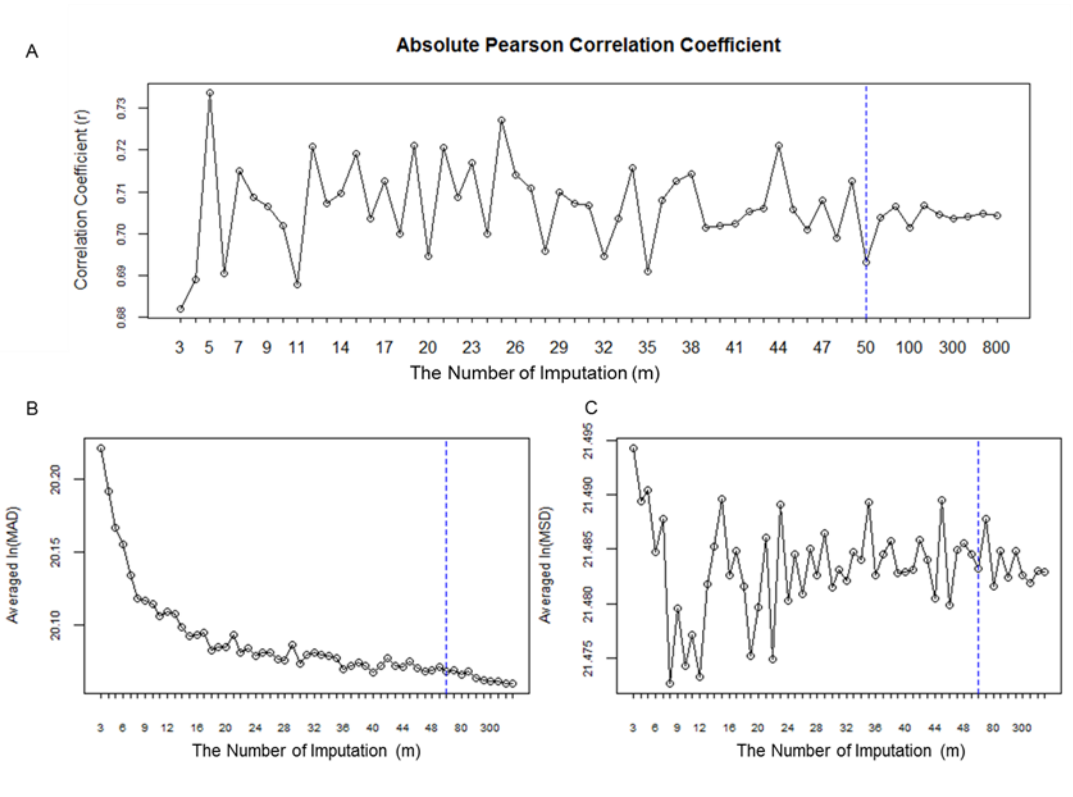

Figure S7. Line charts of absolute Pearson correlation coefficient as well as the averaged ln(MAD) and ln(MSD) in simulated dataset D0(h) with 40% missing value percentage.

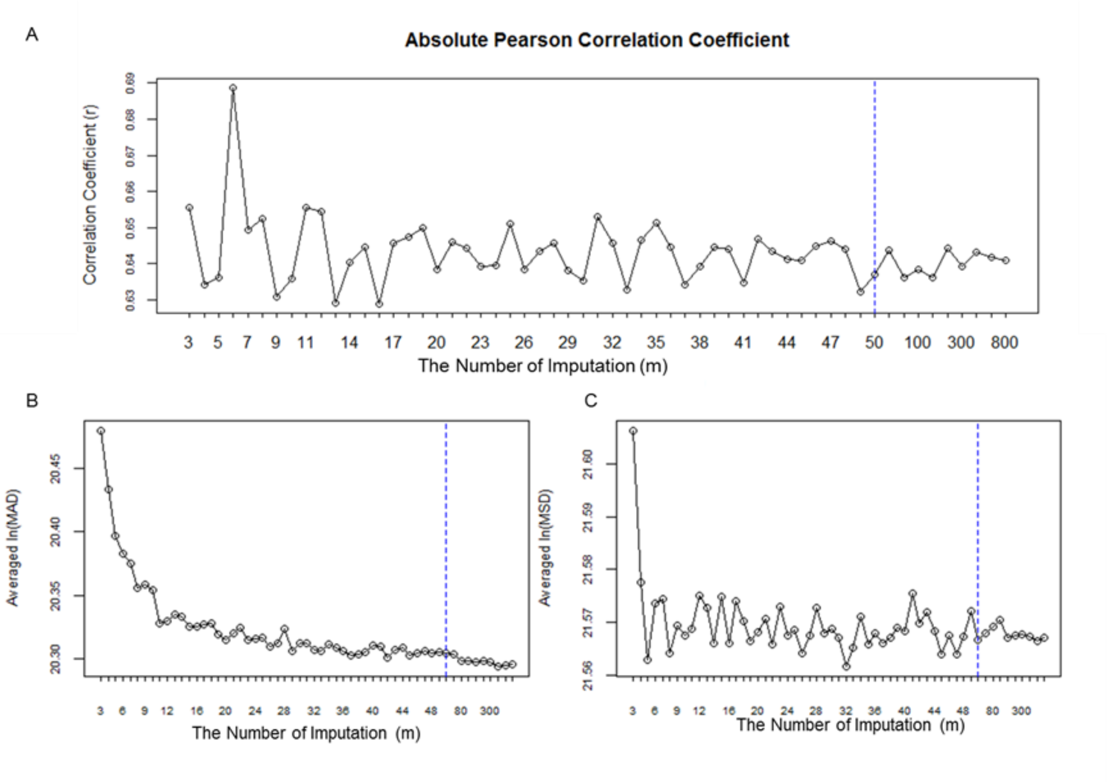

Figure S8. Line charts of absolute Pearson correlation coefficient as well as the averaged ln(MAD) and ln(MSD) in simulated dataset D0(i) with 45% missing value percentage.

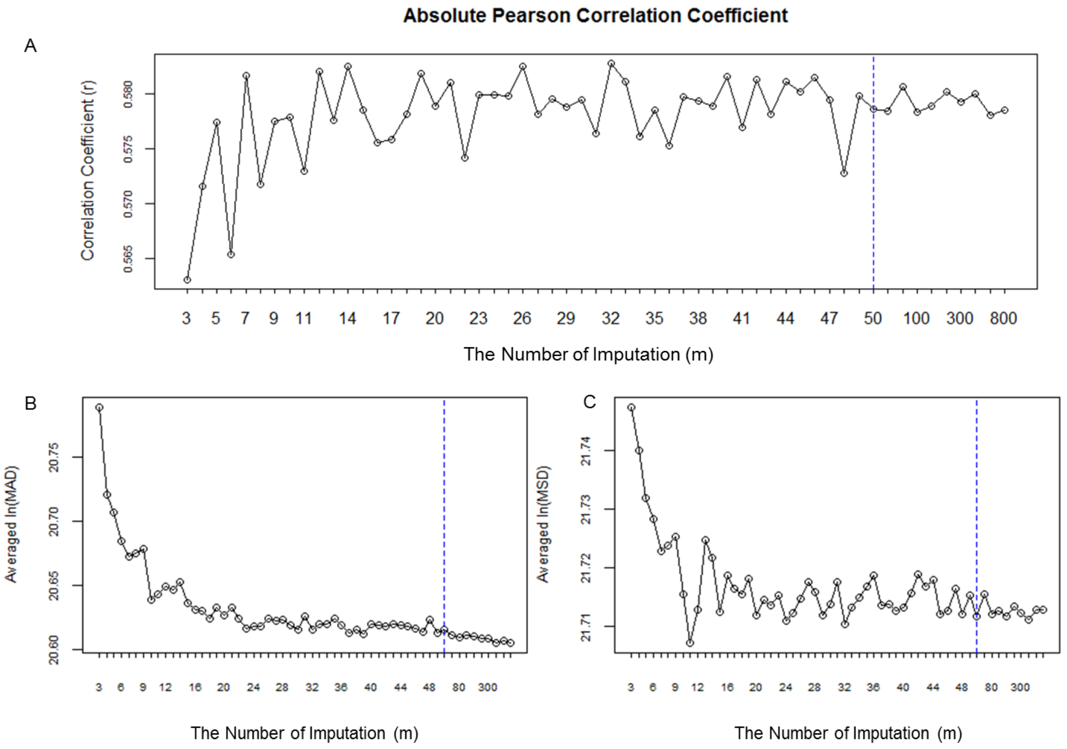

Table S1. Definitions of the indices used to evaluate the performances of DEP detection methods.

Measure	Equation
Sensitivity = Recall (R) = TPR	$\frac{TP}{TP+FN}$
Specificity = TNR = 1- FPR	$\frac{TN}{TN+FP}$
FDR	$\frac{FP}{FP+TP}$
Precision (P)	$\frac{TP}{TP+FP}$
f-score	$\frac{P\times R}{P+R}$
g-score (Geometric Mean Accuracy)	$\sqrt{TPR\times TNR}$
